# Development of a Wireless Computer Vision Instrument to Detect Biotic Stress in Wheat

**DOI:** 10.3390/s140917753

**Published:** 2014-09-23

**Authors:** Joaquin J. Casanova, Susan A. O'Shaughnessy, Steven R. Evett, Charles M. Rush

**Affiliations:** 1 University of Florida, Gainesville, FL 32611, USA; E-Mail: joaquin.casanova@gmail.com; 2 USDA-ARS, P.O. Drawer 10, Bushland, TX 79012, USA; E-Mail: steve.evett@ars.usda.gov; 3 Texas A&M AgriLife Research & Extension, Amarillo Blvd., Amarillo, TX 79109, USA; E-Mail: crush@ag.tamu.edu

**Keywords:** crop stress, image segmentation, irrigation management, maximum expectation algorithm

## Abstract

Knowledge of crop abiotic and biotic stress is important for optimal irrigation management. While spectral reflectance and infrared thermometry provide a means to quantify crop stress remotely, these measurements can be cumbersome. Computer vision offers an inexpensive way to remotely detect crop stress independent of vegetation cover. This paper presents a technique using computer vision to detect disease stress in wheat. Digital images of differentially stressed wheat were segmented into soil and vegetation pixels using expectation maximization (EM). In the first season, the algorithm to segment vegetation from soil and distinguish between healthy and stressed wheat was developed and tested using digital images taken in the field and later processed on a desktop computer. In the second season, a wireless camera with near real-time computer vision capabilities was tested in conjunction with the conventional camera and desktop computer. For wheat irrigated at different levels and inoculated with wheat streak mosaic virus (WSMV), vegetation hue determined by the EM algorithm showed significant effects from irrigation level and infection. Unstressed wheat had a higher hue (118.32) than stressed wheat (111.34). In the second season, the hue and cover measured by the wireless computer vision sensor showed significant effects from infection (*p* = 0.0014), as did the conventional camera (*p* < 0.0001). Vegetation hue obtained through a wireless computer vision system in this study is a viable option for determining biotic crop stress in irrigation scheduling. Such a low-cost system could be suitable for use in the field in automated irrigation scheduling applications.

## 1. Introduction

Scientific irrigation scheduling involves plant, soil or weather based measurements and can be used to effectively reduce water use in agriculture. In an early attempt to manage irrigations, Jensen [1] combined climate and soil water inputs to a computer program to manage irrigation prescriptions. More recently, sensors that monitor plant responses to the environment have been used for crop water management. Jones [2] describes several plant-based measurements for irrigation scheduling, which include sensors that measure tissue water status (tissue and stem leaf water potential) and crop physiological responses (vapor diffusion, canopy temperature, or dendrometry techniques) to water stress. Sensor-based water management can improve crop water use by applying water to crops only when it is needed. Spatial and temporal disease detection can also eliminate unnecessary water applications by terminating irrigations in portions of a field where the crop becomes severely diseased. Sensitivity to plant water- and disease-stress is a major consideration for sensor-based irrigation scheduling. As early as the 1980s, Idso *et al.* [3] and Jackson [4] demonstrated that monitoring crop canopy temperature with infrared (IR) thermometry provided the level of sensitivity necessary to detect crop water stress. Since then there have been numerous studies to document successful irrigation scheduling with IR. At the Bushland, Texas, USDA-ARS Research Laboratory, automated irrigation scheduling of corn, soybean, cotton and sorghum with wired and wireless IR instrumentation has shown to be effective in producing yields that are similar to or better than those irrigated based on direct soil water measurements with a neutron probe [5–8].

Along with IR thermometry, optical remote sensing techniques can be applied for examination of abiotic (e.g., nutrient deficits or salinity) and biotic stresses (disease or pestilence) in wheat. Fitzgerald *et al.* [9] paired reflectance data with the Canopy Chlorophyll Content Index and the two-Dimensional Crop Water Stress Index to remotely detect levels of N and water stress in wheat. Spectral reflectance measurements have also been useful to remotely detect pest infestation or disease. Mirik *et al.* [10] reported that aphid-infested wheat provided higher spectral reflectance measurements in the near-infrared range and a decreased reflectance in the visible range of the spectrum compared with non-infested wheat. Bravo *et al.* [11] demonstrated that spectral reflectance can detect yellow-rust disease in wheat in early spring, using a spectrograph on a pushcart. Wavebands that were useful in disease detection were 543 nm, 630 nm, 750 nm, and 861 nm. Workneh *et al.* [12] were able to track the spread of WSMV over time and space in a large-sized field using spot reflectance measurements from the 555 nm wave band with a hand-held spectral radiometer. The prevalence of disease has a large negative economic impact on winter wheat production in the western United States [13], and remote detection of the disease could improve crop management decisions for optimizing yields and profits.

Another type of remote sensing for crop management is computer vision (CV), which refers to processing and analyzing digital images to determine specific attributes. The goal of the system is to automate processing of a digital image by detecting specific shapes or colors that result in object recognition. The basic components of a CV system typically include an imaging sensor, a processing device (computer or chip) to manage image processing algorithms and a means for data output, *i.e.*, a computer or LCD screen. Imaging sensors can range from expensive charged-couple device (CCD) cameras to low-cost high resolution digital cameras. Atas *et al.* [14] used a CCD camera for aflatoxin detection in chili pepper. Bravo *et al.* [11] describes a multi-sensor platform consisting of a fiber optic spectrograph and multispectral (visible to near infrared range) camera connected to a computer laptop and mounted to a tractor to detect and treat fungal disease in wheat. Xue *et al.* [15] used an inexpensive digital camera with a resolution of only 640 × 480 pixels as part of a machine vision guided agricultural robotic system to independently traverse through a cornfield planted in rows. Contreras-Medina *et al.* [16] describe a smart-sensing CV system comprised of an imaging sensor attached to a hardware signal processing (HSP) unit, high intensity LEDs for illumination, opaque white panels to hold the plant sample, and a computer or LCD screen as the output unit. Because disease in plants is typically manifested by changes in leaf color, malformation of leaf structure, or tissue injury, they were able to characterize a leaf using RGB components to define color attributes corresponding to disease and quantitatively estimate its percent of diseased and necrotic areas.

Automated, quick, and accurate image interpretation is a valuable attribute for agricultural applications. Such applications are quite varied, but include detecting disease in citrus as shown by Pydipati *et al.* [17], identifying nutrient deficiencies in leafy vegetable crops as performed by Story *et al.* [18], and detecting weeds for precision application of herbicides as discussed in Meyer *et al.* [19] and Berge *et al.* [20]. Equally varied are the methods used to provide object recognition from the digital analysis. For example, Meyer and Neto [21] calculated normalized difference vegetative indices from image pixels and used thresholds and Gaussian mixture models for improved classification of plant biomass. Golozarian *et al.* [22] calculated hue values for each pixel within a color digital image and used pre-established thresholds to classify the image into percent vegetation and soil. Hue is an attribute of color that was reported by Wobbecke *et al.* [23] to be an effective and relatively lighting-independent index for image segmentation between different plant species. Laliberte *et al.* [24] used hue to distinguish between senescent and green plants. Ribeiro *et al.* [25] applied genetic algorithms to segment residue and quantify residue cover from images of a cropped field. Camargo and Smith [26] transformed RGB images into different color attributes (hue, I3a, and I3b), and determined the position of local maximums within histograms to segment the image into diseased and healthy regions. Phadikar *et al.* [27] classified diseases from images of rice plants by developing novel algorithms based on the Fermi energy concept to characterize color, shape, and leaf position.

Although there are a number of agricultural CV applications for the detection of nutrient deficiencies, disease or insect-damage, studies concerning CV applications to detect abiotic stresses are limited. In the case of production agriculture, where large-sized fields are planted, continuous sensor measurements to monitor biotic and abiotic stress and the ability to scale the sensors over large areas are key to sustainable irrigation management. Remote detection of crop water stress would allow for improved irrigation scheduling by providing irrigation only when needed, while detection of disease could aid in signaling termination of irrigation where yield potentials will be minimal. Low-cost computer vision instruments and wide availability of wireless digital cameras make CV systems a potentially scalable tool in the remote detection of abiotic and biotic crop stresses. Vegetation hue from digital images may be useful for detecting disease and water stress, while estimates of vegetation cover are potentially useful for qualifying measurements of IR temperature [28]. This paper describes a low-cost compact wireless CV system that performs image segmentation onboard the sensor's microprocessor; using hue determines the impacts of wheat streak mosaic virus (WSMV) infection and crop water stress on computer vision-derived hue and vegetation cover; and compares hue and disease detection using this system with that resulting from use of a camera with greater image and pixel color resolution. Advantages of these economical wireless compact image-sensing instruments over standard digital cameras are the ability to deploy multiple sensors, automated image acquisition and analysis, and retrieve critical information remotely.

## 2. Experimental Section

This section explains the field experiments for wheat over the 2011 and 2013 seasons, the algorithmic details for the image processing, and the two imaging systems, one of which was a wireless computer vision system designed specifically for this study.

### 2.1. Field Experiments

**Figure 1.**
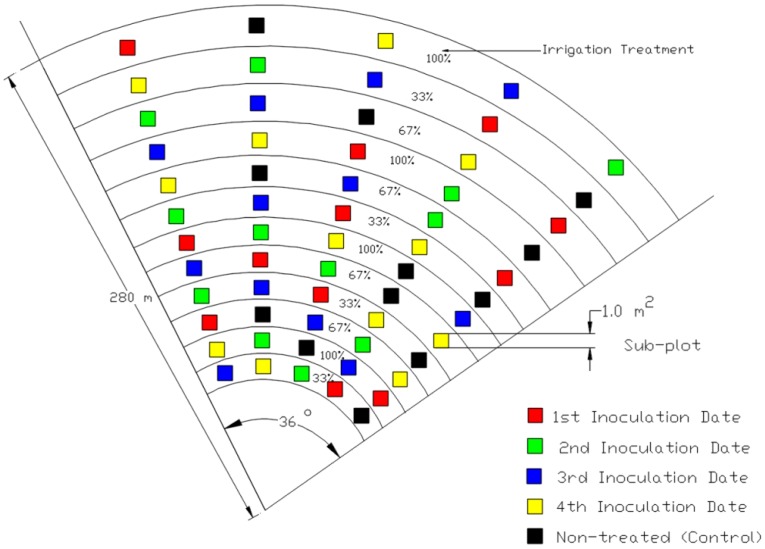
Wheat experiment plot design layout in 2011.

The first season (2011) was an evaluation of the computer vision algorithms using a conventional digital camera with images processed later on a desktop. Field experiments were conducted at the USDA ARS Conservation and Production Research Laboratory, Bushland, Texas, USA (35°11′N, 102°06′W, 1170 m above mean sea level). Winter wheat, WSMV susceptible cultivar Karl 92, was planted under a six-span center pivot irrigation system on 29 November 2010 at a rate of 78 kg·ha^−1^. Sixty treatment plots (4 m × 4 m) were arranged within a 36° sector in a split-plot design with whole plots receiving irrigation amounts of 100%, 67%, and 33% replenishment of soil water depletion to field capacity (designated I100%, I67%, and I33%). Subplots were comprised of 12 control plots (non-inoculated) and those receiving inoculations of WSMV on a given date (17 March, 1 April, 15 April, and 2 May 2011). Irrigation treatment levels were applied concentrically and replicated radially four times (Figure 1). Inoculation dates were staggered to establish varying levels of disease severity (with earlier dates expected to result in more severe levels). The actual infection status was determined by enzyme-linked immunosorbent assay (ELISA). Plants were selected randomly from each subplot for the assay using methods by Workneh *et al.* [12]. This evaluation provided a qualitative analysis of disease incidence. Not all plots that were inoculated became infected, and because it was not feasible to test all plants or all leaves of each plant within each subplot for the virus, digital imagery to detect biotic stress was investigated. Digital images were taken at 10:30 am through 1:30 pm CST at a nadir view with the camera lens pointed downwards at a distance perpendicular to the ground over an aluminum wrapped target placed within the subplot, 0.6 m × 0.6 m. The focal length was adjusted manually to locate the target within the viewfinder of the camera. Images were taken over 60 plots each day on 10 May, day of year, (DOY) 130 and 17 May (DOY 137) in 2011 with a RGB digital camera (model IS-1, Fuji, Edison, NJ, USA; the mention of trade names of commercial products in this article is solely for the purpose of providing specific information and does not imply recommendation or endorsement by the U.S. Department of Agriculture). The lens was equipped with a UV-IR cut filter to block wavelengths outside the visible range since the camera was sensitive to wavelengths ranging from 400 to 900 nm. The lens was filtered because unfiltered, in bright sunlight and with automatic exposures, the R channel will saturate due to NIR and IR wavebands. One image was taken per plot, covering an area of approximately 1 m^2^ in the JPEG format at a resolution of 1600 pixels × 1200 pixels with 8 bits per each of the three color channels. Images were cropped to remove non-soil or non-vegetation objects, such as shoes. The resulting data set had a total of 120 samples representing three irrigation treatment amounts and the two inoculation levels (0- control plots, and 1- inoculated).

During the 2013 season, the purpose was to evaluate the performance of the wireless computer vision sensor (described in Section 2.3) in comparison to the Fuji. The 2013 field experiment had a similar layout as 2011, with differential levels of water stress applied through different irrigation amounts and differing dates for WSMV inoculation (14 March, 2 April, 16 April, and 30 April 2013) to simulate differences in infection level. However, due to the time-consuming nature of testing both camera systems, we only examined two plots, one which was fully irrigated and healthy, and another which was fully irrigated and infected with WSMV. Four images were taken of each plot, two images each from both the Fuji and the wireless computer vision system on 11days (DOY 74, 80, 88, 94, 109, 116, 123, 130, 136, 148, 157) throughout the season at 1:00 pm through 3:00 pm CST. Blurry images or images not well centered over the target were discarded. The resulting data set had a total of 58 samples representing the two inoculation levels and the two cameras. Due to seasonal variations, the development of the wheat crop and infection by WSMV lagged in 2011 (an exceptional drought year with annual rainfall totaling 136 mm) as compared with 2013 where annual precipitation was 80% of the historical average (470 mm). This retardation was the reason for the large difference in DOY between image acquisition for 2011 and 2013. Images were acquired the day after the first inoculation and continued until senescence. Symptom expression or chlorosis of plants within the subplot provided a visual indication of WSMV infection. Symptom expression is highly dependent on temperature, so the time it took for symptoms to be expressed varied between years and within each season.

### 2.2. Image Processing Algorithms

The image processing algorithm consisted of three main steps, and was implemented in MATLAB. First, the RGB images of the crop were transformed into hue. Hue is part of the hue, saturation, and value (HSV) colorspace and uses red, green, and blue components of pixel values (the R, G, and B reflectances), scaled by the sum of R, G, and B, as in Equation (1) where r_i_ is the scaled R value for pixel i, g_i_ is the scaled G value for pixel i and b_i_ is the scaled B value for pixel i, resulting in scaled reflectances r_i_, g_i_ and b_i_, respectively:

(1) $$r_{i}=\frac{R_{i}}{R_{i}+G_{i}+B_{i}},g_{i}=\frac{G_{i}}{R_{i}+G_{i}+B_{i}},b_{i}=\frac{B_{i}}{R_{i}+G_{i}+B_{i}}$$

Hue (*x*) is calculated as in Golzarian *et al.* [22]:

(2) $$x_{i}=\text{arctan}\left(\frac{\sqrt{3}\left(g_{i}-b_{i}\right)}{\left(r_{i}-g_{i}\right)+\left(r_{i}-b_{i}\right)}\right)$$

where arctan is the 360° arctangent function. While value was useful for discriminating lit and shadowed components of the image in preliminary tests, our goal here was discrimination of soil and vegetation components, which was effectively accomplished by hue alone.

Second, the images were analyzed by way of the distribution of hue values for a given image. The probability distribution of hue values for each image (*f*) was assumed to be a mixture of two Gaussians [29], corresponding to the two classes of interest, soil (*s*) and vegetation (*v*):

(3) $$f=\frac{p_{s}}{\sqrt{2\pi }\sigma_{s}}\exp\left(\frac{{\left(x-\mu_{s}\right)}^{2}}{2\sigma_{s}^{2}}\right)+\frac{p_{v}}{\sqrt{2\pi }\sigma_{v}}\exp\left(\frac{{\left(x-\mu_{v}\right)}^{2}}{2\sigma_{v}^{2}}\right)$$

The expectation maximization (EM) algorithm, an iterative optimization method discussed by Moon [30], was applied to fit the mixture of the two Gaussian distributions of hue for each Fuji image. Essentially the EM algorithm maximizes the log-likelihood of the Gaussian parameters providing estimates of mean hue (μ*_j_*), standard deviation of hue (σ*_j_*), and prior probability (*p_j_*) of hue for each class *j*. The mean vegetation hue (μ*_v_*) and vegetation cover (*p_v_*) for each image were determined from the EM algorithm with soil and vegetation as the two classes.

Alternatively, the mean vegetation hue of an image can be determined using threshold-based image segmentation in which the hue for each pixel is compared with an established hue threshold. For this method, a hue value greater than the threshold is to be classified as vegetation, and one lower is to be classified as soil. After analyzing Fuji images using the EM algorithm, the optimal hue threshold between soil and vegetation was determined by using the maximum likelihood criterion [29]. The threshold was determined by solving Equation (4) for the hue value x by substituting the Gaussian parameters (μ_v_, μ_s_, σ_s_, σ_v_, ρ_v_, ρ_s_) determined from Fuji images with the EM algorithm:

(4) $$\frac{{\left(x-\mu_{s}\right)}^{2}}{2\sigma_{s}^{2}}+\frac{{\left(x-\mu_{v}\right)}^{2}}{2\sigma_{v}^{2}}=\text{ln}\left(\frac{\sigma_{v}p_{s}}{\sigma_{s}p_{v}}\right)$$

**Figure 2.**
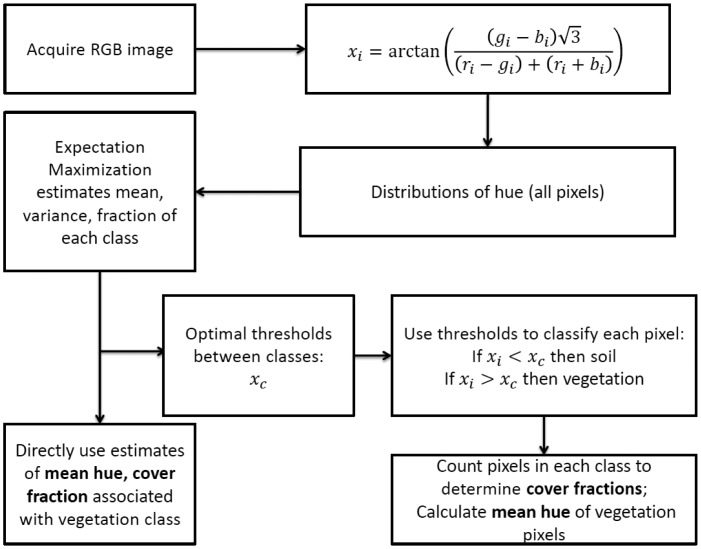
Flow chart of image processing algorithms for Fuji images.

Pixels with hue values greater than x were classified as vegetation, those less than were classified as soil The mean vegetation hue for each image was then calculated as the mean hue value of pixels classified as vegetation. The ratio of pixels classified as vegetation to total pixels in the image was considered the canopy cover fraction. Likewise, the mean soil hue was the mean hue of pixels classified as soil, and the soil fraction was the fraction of pixels in the image classified as soil. Figure 2 shows a flow chart of the image processing techniques.

To establish the significance of the effects of disease stress and water stress on computer vision metrics during the first season, μ*_v_* and *p_v_* for each Fuji image was determined (using a MATlab implementation of the EM algorithm) on a desktop computer, and both the EM-determined and the threshold-determined mean vegetation hue were tested on images of plots that were fully and deficit irrigated; and images of plots that were inoculated with WSMV. Effects of infection, irrigation and DOY of inoculation, and their interactions were analyzed in R by linear mixed effects using repeated measures in time and a compound symmetric covariance structure. To compare the performance of the conventional computer vision and wireless computer vision system, we examined the significance of the effects of disease stress on both vegetation hue and cover fraction for each sensor, using a one-sided paired t-test. When using the wireless computer vision instrument, we used the threshold-based method (explained in Section 2.3) to segment the image into vegetation and soil. This method enabled the wireless CV system to process the image even with low computational capabilities. The threshold was determined using the same analysis of Fuji images as described above.

### 2.3. Wireless Computer Vision Sensor

**Figure 3.**
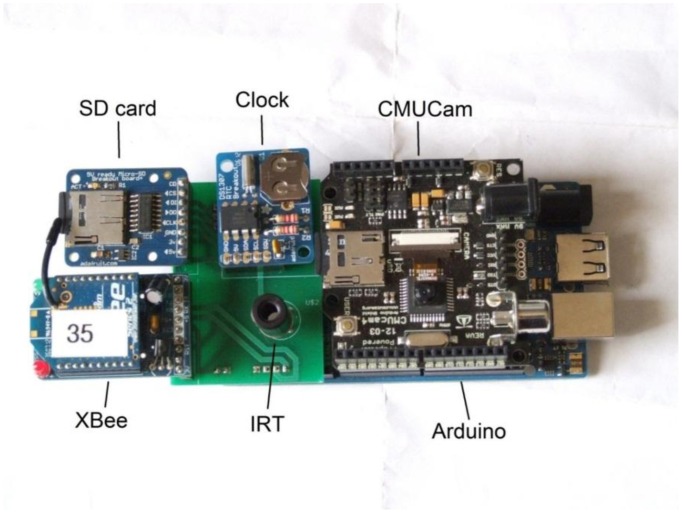
Wireless computer vision sensor.

The procedure of manually taking images in the field with a consumer-level digital camera and later using the EM algorithm on a PC to analyze the information for use in irrigation scheduling is impractical for routine irrigation scheduling. Thus, it is worthwhile to consider how computer vision techniques could be used in the field. Such a system must be able to take RGB images of sufficient resolution for processing to distinguish between plant, soil and other surfaces. Additionally, there should be a microprocessor with enough computational power to calculate the pixel hues from RGB values and the mean vegetation hue of the image. Processor speed is not as critical as memory, because irrigation systems move slowly and changes in vegetation stress are gradual. Finally, an ideal sensor could wirelessly transmit time-stamped hue data to an irrigation scheduling controller. A system meeting these criteria was built using the Arduino Mega ADK [31] as the microprocessor, combined with the CMUCAM4 camera and image processing system [32] and an IR cutoff filter to avoid Red channel saturation in bright sunlight. For speed and memory purposes, we chose a resolution of 120 by 160 pixels, less than the maximum of 480 × 640. The color resolution was 5 bits for red and blue and 6 bits for green. Additionally, an XBee series 2 RF module (Digi International, Minnetonka, MN, USA.) was included for wireless communications and a real time clock for timestamps, as well as a micro SD card for data storage. Figure 3 shows the system. To acquire images in the field, the CV sensor was placed inside of a plastic housing with openings for the camera lens and IRT sensor. Since the CV instrument did not have a viewfinder, the camera was centered above the aluminum target at a vertical height of 1.2 m (since the imaging sensor field-of-view was approximately 25°), at a downward looking angle. The vertical height was measured each time prior to the acquisition of the image. For each plot, images from the CV instrument were acquired immediately after the Fuji images were taken.

**Figure 4.**
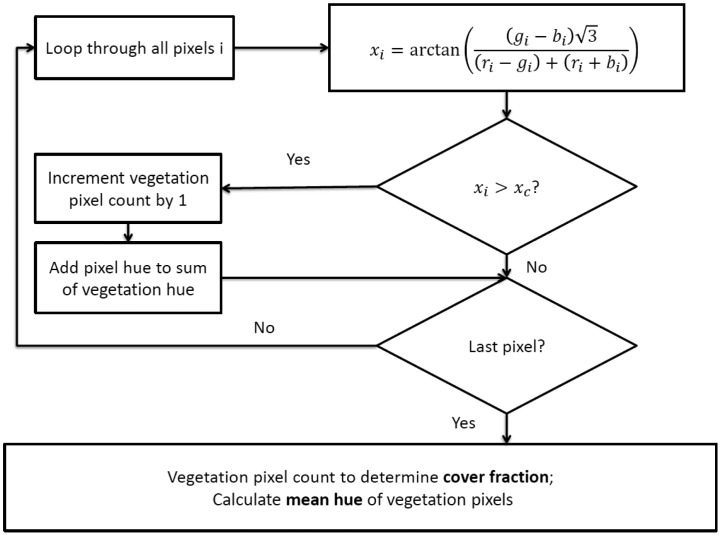
Flow chart of image processing algorithms for wireless computer vision sensor.

While the full EM algorithm would exceed the computational abilities of the Arduino, the images could be analyzed easily using a predetermined threshold. The threshold used on the wireless system was initially set at the same value as determined from the 2011 Fuji images (hue = 25), and then adjusted to minimize differences in fraction of vegetation between the Fuji and wireless computer vision system. This analysis was done by inspection of the segmented images and hue distributions from the wireless system to ensure that images were properly segmented and to achieve estimates of cover fraction similar to the estimates from the Fuji images taken of the same area. A single hue threshold value of 45 was finally used to segment the images taken with the wireless CV system. To remain within the memory constraints of the Arduino (8 kb RAM and 256 kb flash), the images must be processed pixel by pixel. The hue is calculated for each pixel, then the pixel is classified as vegetation or soil. If the pixel is vegetation, the number of vegetation pixels is incremented by one, and the hue of the pixel is added to a running sum of vegetation hue. Once all of the pixels have been looped through, the average vegetation hue and the fraction of vegetation cover are calculated. The timestamp, vegetation hue, and fraction of vegetation cover were written to an SD card and could be sent wirelessly using the XBee. Figure 4 shows a flowchart of the image processing on the wireless computer vision sensor.

## 3. Results and Discussion

### 3.1. 2011 Season

**Figure 5.**
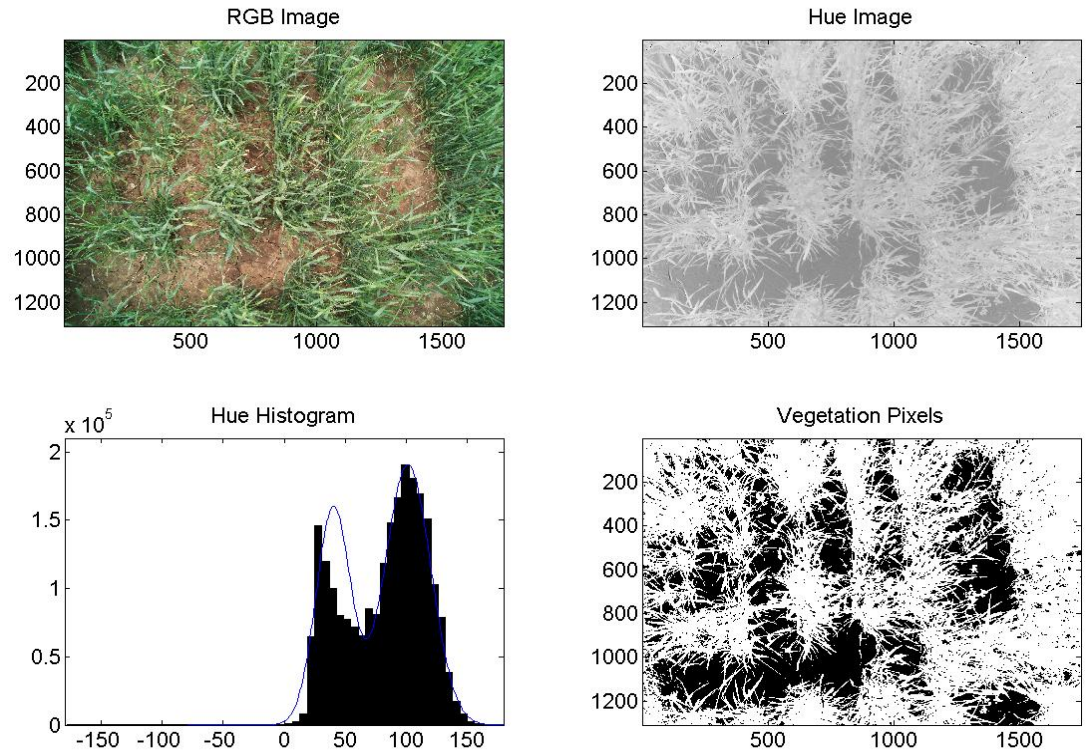
Example image analysis for wheat, DOY 137, 2011, healthy, 100%. Axes on images are in pixels. Pixels classified as vegetation are shown in white (lower right).

**Figure 6.**
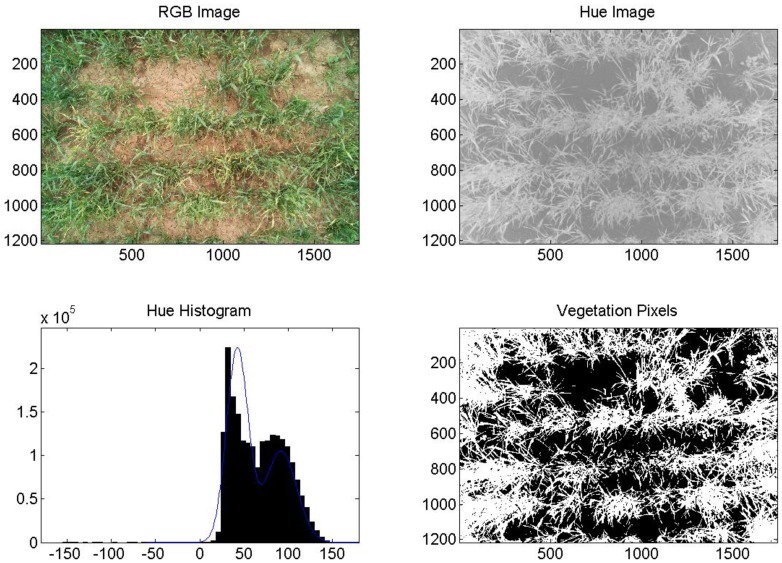
Example image analysis for wheat, DOY 137, 2011, diseased, 100%. Axes on images are in pixels. Pixels classified as vegetation are shown in white (lower right).

The commercial camera (Fuji), successfully segmented images of healthy, fully irrigated, deficit irrigated, and diseased wheat into areas of vegetation and soil. An example of healthy (non-inoculated, irrigation level = 100%) wheat is shown in Figure 5. An image of diseased and fully irrigated wheat is shown in Figure 6. In addition to the RGB images, images made from hue values, histograms exhibiting binomial distribution (soil and vegetation), and images segmented into pixels of vegetation (white) and soil (black) are shown to demonstrate the technique. Of the images acquired over diseased wheat, 67% were correctly segmented into vegetation and soil. However, we did determine that senesced wheat (not shown) could be mistaken for soil because its hue values are similar.

**Figure 7.**
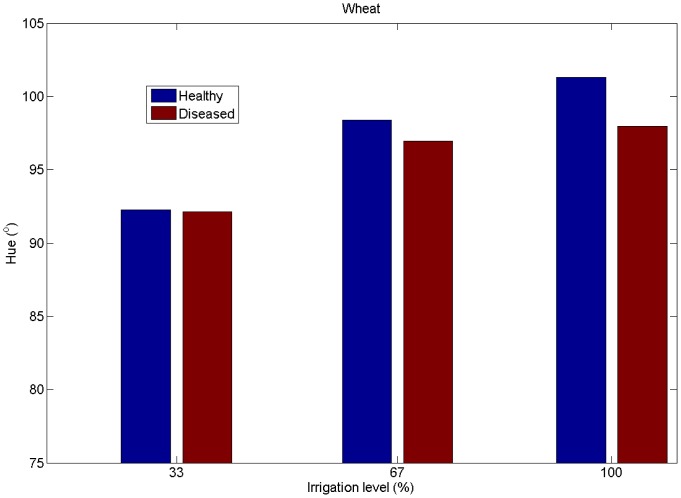
EM-determined seasonal averages of vegetation hues for different treatments in 2011.

We then analyzed the impact of the main effects of WSMV infection, irrigation level, and DOY of inoculation on mean vegetation hue. Results of the linear mixed model test are given in Table 1. Irrigation level and DOY had a significant effect (α = 0.05) on vegetation hue, while infection had a nearly significant effect (*p* = 0.056) when using EM-estimated mean hue. In general, the mean vegetation hue grouped by irrigation treatment level was less for diseased wheat as compared with healthy wheat. The differences were more pronounced in the well-irrigated wheat (treatment levels 100% and 67%) (Figure 7). The greatest difference in mean hue occurred between the healthy 100% irrigation treatment and the diseased 33% irrigation treatment (9.08), but the difference between healthy wheat irrigated at 100% and 33% was similar at 8.08. This indicates that the CV algorithm cannot easily distinguish between healthy and diseased wheat irrigated at a deficit level of 33% of full irrigation. The hue values were likely confounded by the high percent of soil background. However, at well-irrigated levels (100% and 67%), the CV system can distinguish between healthy and diseased wheat.

None of the interaction terms had a significant effect on mean hue. Using the threshold determined hue (value of 25) to segment images into vegetation and soil taken by the commercial-grade camera resulted in p-values that were larger than when segmenting images with the EM-based hue, but the significance of the results were similar (Table 2), with the exception of the effect of infection (*p*-value of 0.0893). This difference between the two hue estimates, most noticeable in sensitivity to infection status, suggests that there would be some benefit to implementing the full EM algorithm in the field, as opposed to a simple threshold technique. Additionally, doing so would help minimize errors due to incorrect segmentation. For vegetation cover, both EM-estimated values and threshold-estimated values were significantly impacted by irrigation levels, but not infection status, likely due to the low severity of the infection. That is, the infection was severe enough to cause yellowing, but did not affect the cover fraction appreciably.

### 3.2. 2013 Season

**Figure 8.**
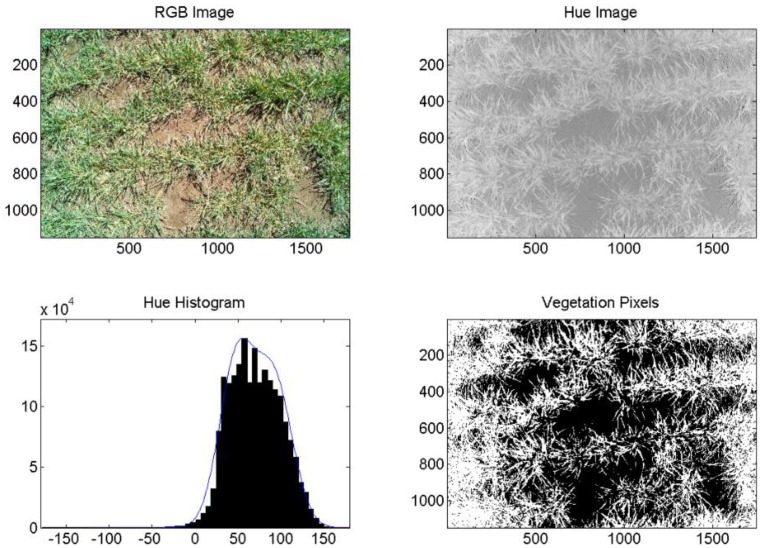
Example of Fuji image analysis for wheat, DOY 88, 2013, diseased, 100%. Axes on images are in pixels. Pixels classified as vegetation are shown in white (lower right).

**Figure 9.**
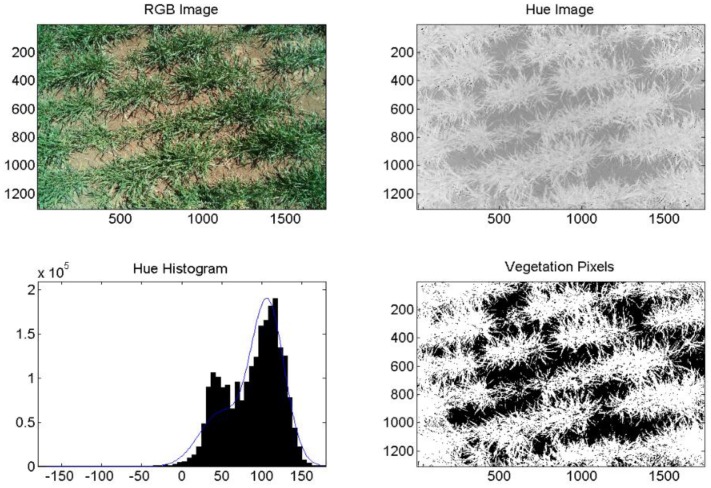
Example of Fuji image analysis for wheat, DOY 88, 2013, healthy, 100%. Axes on images are in pixels. Pixels classified as vegetation are shown in white (lower right).

**Figure 10.**
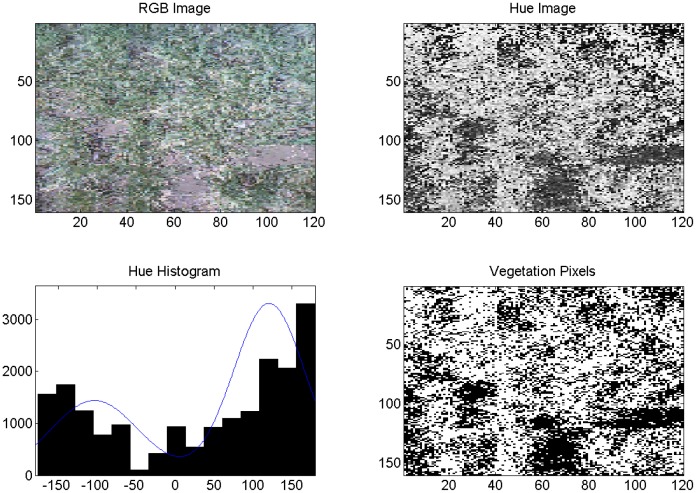
Example of wireless computer vision image analysis for wheat, DOY 88, 2013, diseased, 100%. Axes on images are in pixels.

**Figure 11.**
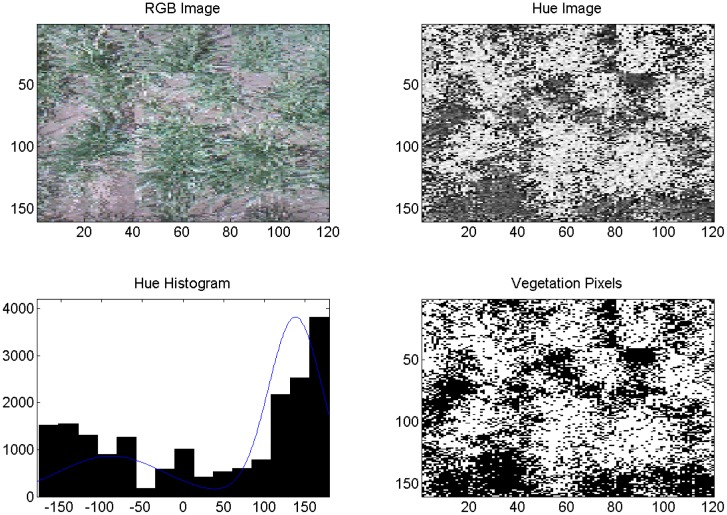
Example of wireless computer vision image analysis for wheat, DOY 88, 2013, healthy, 100%. Axes on images are in pixels.

Both cameras were used on the same DOY in 2013 to acquire and segment digital images of healthy and diseased wheat and percent vegetation and soil. In this case, the irrigation treatment level was the same. Figure 8 is an image of diseased, fully irrigated wheat (100%) and Figure 9 is an image of healthy, fully irrigated wheat (100%), both taken on DOY 88, using the Fuji camera and MATLAB processing. The hue distribution in Figure 8 is not binomial, which is indicates that hue values for vegetation and soil were similar to one another. This may be due to severity level of WSMV as compared with the diseased wheat shown in Figure 6 taken in 2011. Figures 10 and 11 show the corresponding images from the wireless computer vision system. Again, images made from hue values, histograms exhibiting distribution of soil and vegetation pixels, and images segmented into pixels of vegetation (white) and soil (black) are shown in addition to the RGB images. Both sets of images were processed using thresholds, because of the computational limitations of the wireless system. Images were segmented into vegetation and soil using the threshold-based method with manually selected hue thresholds of 25 for the Fuji images and 45 for the wireless CV images. Figures 10 and 11 show that even though the resolution was considerably reduced using the CMUCAM, vegetation and soil were still distinct. In the CMUCAM images, there were some discontinuities, due to the adaptation of the image processing algorithms to the limited RAM of the Arduino. The image was taken and processed in 40 pixel × 40 pixel chunks to remain within the 8KB of SRAM. Additionally, the hue values were different from the Fuji, due to differences between the cameras' color resolution (16 bit for the CMUCAM, 24 bit for the Fuji) and firmware.

Once the images were segmented into vegetation and soil, mean vegetation hue values for each image were analyzed. Results of the one-sided paired t-tests (using JMP10.0.0, SAS Institute, Inc., Cary, NC, USA) comparing healthy and diseased vegetation hues from both computer vision systems are given in Table 3. Both systems' measurements of hue were significantly affected by the disease status. The differences in means between hue values for the Fuji camera and wireless CV system were 16.89 and 4.52, respectively.

Using a hue threshold of 98 for the Fuji and 122.5 for the CV instrument, where images with a mean vegetation hue value below the threshold are classified as disease, and those above are classified as healthy, images taken by the FUJI were correctly classified 82% of the time, while those from the CV instrument were correctly classified 68% of the time. These threshold values were calculated as the upper control limit for mean hue values over diseased plots (Equation (5)):

(5) $$\text{UCL}={\bar{X}}_{w}+\frac{\hat{k}\hat{\sigma }}{\sqrt{n_{i}}}$$

where *X̄_w_* is the weighted average of the subgroup means (non-infected, infected vegetation hue), *k̂* is the sigma multiplier, *σ̂* is the estimated standard deviation, and n_i_ is the sample size (22 and 36 for the Fuji and wireless images, respectively).

Although the Fuji was more sensitive to differences in hue, due to its greater spatial and color resolution, the lower-resolution wireless system was also able to detect differences in hue over healthy and diseased wheat, albeit with lesser accuracy. Likewise, both systems detected a significant difference in cover fraction.

## 4. Conclusions

Knowledge of crop abiotic and biotic stress is important for efficient irrigation and disease management. This is especially the case under variable rate irrigation control, where irrigation to diseased areas in a field could be terminated, while irrigation could continue to be applied to unaffected areas within the field. In this study, we initially used a commercial grade digital camera and laptop to segment images using an expectation maximization (EM) algorithm applied to the hue distribution of digital images over healthy and diseased wheat containing vegetation and soil. Mean hue values for vegetation and soil that were derived from the EM algorithm, were used to estimate percent vegetative cover within an image. Secondly, we developed a wireless computer vision instrument and used a pre-established hue threshold to segment digital images (onboard the imaging system) into percent vegetation and soil. Image processing was completed onboard the wireless CV instrument. We also demonstrated that water stress (deficit irrigation levels) significantly lowered vegetation hue value at *p* < 0.05, and that WSMV disease significantly lowers hue at *p* = 0.10 level. During the 2011 growing season, the algorithm was tested using images from a commercial camera and processed later on a desktop. The data from this season indicated that mean vegetation hue determined by EM was significantly impacted by wheat disease stress (infected by WSMV) and water stress (irrigation level). Vegetation cover showed significant effects from irrigation but not infection, due to relatively low disease severity. During the 2013 season, a modified algorithm was implemented onto a real-time wireless computer vision sensor using a fixed threshold rather than the full EM algorithm. The computer vision sensor was tested on one healthy and one diseased plot, over the course of the growing season, along with the commercial camera. Both systems were able to detect the difference in hue and vegetation cover between healthy and diseased wheat. This demonstrated that the computer vision method could be used for detection of crop stress, and that the wireless computer vision sensor was capable of detecting differences between healthy and diseased wheat at higher irrigation levels, despite having less resolution. Future computer vision systems should have greater color and spatial resolution, and computational power to improve accuracy of disease detection and sensitivity of the instrument by implementing a full EM algorithm. Further research is required to determine hue thresholds for disease severity that would establish support for continued irrigations (mild disease) or termination (severe disease). In addition, further work is need to integrate multiple CV instruments along a pivot lateral and determine the efficacy of hue thresholds for triggering irrigations and estimating percent canopy cover at the field-scale level.

## Figures and Tables

**Table 1.** Tests of fixed effects for 2011 wheat EM-based hue and cover fraction (N = 120).

**Effect**	***P*-Value (hue)**	***P*-Value (cover)**
Infection	0.0562	0.8325
Irrigation	0.0004	0.0009
DOY	0.0002	<0.0001
Infection × Irrigation	0.4158	0.8498
Infection × DOY	0.9946	0.7697
Irrigation × DOY	0.1521	0.0113
Infection × Irrigation × DOY	0.7479	0.8437

**Table 2.** Tests of fixed effects for 2011 wheat threshold-based hue and cover fraction (N = 120).

**Effect**	***P*-Value (hue)**	***P*-Value (cover)**
Infection	0.0893	0.8487
Irrigation	0.00043	0.0009
DOY	<0.0001	<0.0001
Infection × Irrigation	0.4590	0.8546
Infection × DOY	0.9855	0.7662
Irrigation × DOY	0.3108	0.0019
Infection × Irrigation × DOY	0.7684	0.7898

**Table 3.** Paired t-test for 2013 hue for infected *vs*. healthy wheat and cover fraction (N = 58).

**Camera**	**Difference in hue Means (*P*-Value)**	**Difference in Cover Fraction (*P*-Value)**
Fuji	16.89 (<0.0001)	0.23 (<0.0001)
Wireless	4.52 (0.0014)	0.08 (<0.0001)
